# Hypofractionated radiotherapy with simultaneous tumor bed boost (Hi-RISE) in breast cancer patients receiving upfront breast-conserving surgery: study protocol for a phase III randomized controlled trial

**DOI:** 10.1186/s13014-024-02449-y

**Published:** 2024-05-27

**Authors:** Kairui Jin, Jurui Luo, Xiaoli Yu, Xiaomao Guo

**Affiliations:** 1 Department of Radiation Oncology, Department of Oncology, Shanghai Medical College, Shanghai Clinical Research Center for Radiation Oncology, Shanghai Key Laboratory of Radiation Oncology, Fudan University Shanghai Cancer Center, Fudan University, 270 DongAn Road, Shanghai, 200032 China; 2 Department of Radiation Oncology, Ren Ji Hospital, Shanghai Jiao Tong University School of Medicine, Shanghai, 200127 China

**Keywords:** Hypofractionated radiotherapy, Simutaneous integrated boost, Regional node irradiation, Breast cancer, Randomized controlled trial

## Abstract

**Background:**

The effectiveness and safety of moderately hypofractionated radiotherapy (HFRT) in patients undergoing breast-conserving surgery (BCS) has been demonstrated in several pivotal randomized trials. However, the feasibility of applying simultaneous integrated boost (SIB) to the tumor bed and regional node irradiation (RNI) using modern radiotherapy techniques with HFRT needs further evaluation.

**Methods:**

This prospective, multi-center, randomized controlled, non-inferiority phase III trial aims to determine the non-inferiority of HFRT combined with SIB (HFRTsib) compared with conventional fractionated radiotherapy with sequential boost (CFRTseq) in terms of five-year locoregional control rate in breast cancer patients undergoing upfront BCS. A total of 2904 participants will be recruited and randomized in a 1:1 ratio into the HFRTsib and CFRTseq groups. All patients will receive whole breast irradiation, and those with positive axillary nodes will receive additional RNI, including internal mammary irradiation. The prescribed dose for the HFRTsib group will be 40 Gy in 15 fractions, combined with a SIB of 48 Gy in 15 fractions to the tumor bed. The CFRTseq group will receive 50 Gy in 25 fractions, with a sequential boost of 10 Gy in 5 fractions to the tumor bed.

**Discussion:**

This trial intends to assess the effectiveness and safety of SIB combined with HFRT in early breast cancer patients following BCS. The primary endpoint is locoregional control, and the results of this trial are expected to offer crucial evidence for utilizing HFRT in breast cancer patients after BCS.

**Trial registration:**

This trial was registered at ClincalTrials.gov (NCT04025164) on July 18, 2019.

**Supplementary Information:**

The online version contains supplementary material available at 10.1186/s13014-024-02449-y.

## Background

Breast cancer is the most common cancer in women worldwide, with an estimated 420,000 new cases in China in 2020. In the management of early-stage breast cancer, breast-conserving surgery (BCS) has been the standard approach [1]. Whole breast irradiation (WBI) after BCS reduces local recurrence by 50%∼75% and improves long-term survival outcomes of breast cancer patients [2]. Multiple prospective trials have demonstrated that BCS, combined with radiotherapy, could yield similar survival outcomes to mastectomy [3–6]. Previously, conventional WBI was performed with a fractionation of 50 Gy in 25 fractions (2 Gy per fraction). However, since the 2000s, hypofractionated WBI has emerged as a viable alternative. This approach delivers 39–42.9 Gy in 13–16 fractions (2.67–3.3 Gy per fraction) and has been proven equally effective and safe as conventional fractionation in several pivotal randomized studies [7–10]. Therefore, the American Society for Radiation Oncology (ASTRO) recommended hypofractionated WBI of 40 Gy in 15 fractions or 42.5 Gy in 16 fractions with sequential boost to the tumor bed as continued treatment after BCS in their 2018 guidelines [11].

Additional boost to tumor bed was proved to further reduce the local recurrence rate, especially in patients younger than 50 years and in patients with high grade invasive ductal carcinoma [12, 13]. The standard tumor bed boost regimen was 10 ∼ 16 Gy in 5 ∼ 8 fractions sequential to WBI [11]. During the last decades, dose escalation to the tumor bed by using the simultaneously integrated boost (SIB) technique has been proposed. Compared with sequential boost, SIB shortens the treatment time by 1 week and is superior in terms of radiobiology and dosimetry [14, 15]. However, due to the lack of high-level evidence, SIB is not routinely recommended in clinical practice other than in clinical trials. Recently, a German trial concerning normofractionated WBI with SIB (IMRT-MC2 trial) demonstrated that intensity modulated radiation therapy (IMRT) with SIB had non-inferior two-year local control and cosmetic results when compared to three-dimensional conformal radiotherapy with sequential boost [16]. The IMPORT HIGH trial is a phase III randomized trial evaluating hypofractionated concurrent boost integrated to WBI of 36 Gy in 15 fractions. Recently, results of this study revealed non-inferiority of a 48 Gy-SIB compared to sequential boost in terms of local control and incidence of breast induration [17]. Therefore, despite becoming a trend in the hypofractionation era, the safety and effectiveness of SIB still need further evidence to be confirmed.

In addition, regional node area was not regularly irradiated in previous trials. 7%∼21% patients of the START trials received regional node irradiation (RNI). A follow-up was done after ten years, and no more arm and shoulder symptoms were observed in the HFRT group. However, these studies did not include internal mammary node (IMN) in the planning target volume. Therefore, the feasibility of hypofractionated RNI (including IMN irradiation) needs to be further investigated [18]. Based on the concerns mentioned above, it is necessary to reevaluate the effectiveness and safety of HFRT with SIB in BCS patients in the context of modern treatment strategies. A phase II trial of hypofractionated WBI (HF-WBI) combined with SIB in early breast cancer patients was conducted at our center, the results of which showed that HF-WBI and SIB could yield satisfactory cosmetic outcomes with mild toxicity. No patient developed severe radiodermatitis and the incidence of excellent/good breast cosmesis was 81.8% [19]. On the foundation of the results from our phase II trial and previous studies, a phase III multi-centered, randomized controlled trial was initiated to investigate the local control following normofractionated WBI with sequential boost (CFRTseq) versus moderately hypofractionated WBI with SIB (HFRTsib) with or without RNI in breast cancer patients after BCS.

## Methods

### Study design

**Fig. 1:**
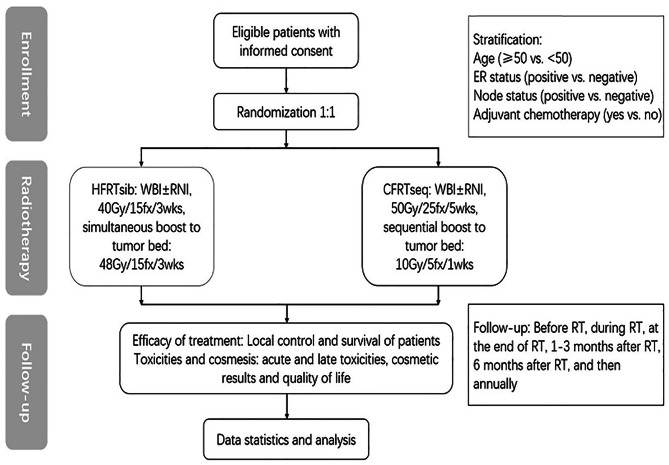
Schematic overview of study design. HFRTsib, hypofractionated radiotherapy with simultaneously integrated boost; CFRTseq, conventional fractionated radiotherapy with sequential boost; WBI, whole breast irradiation; RNI, regional node irradiation; ER, estrogen receptor; RT, radiotherapy

This is a prospective randomized phase 3 study with two arms. The standard regimen is CFRTseq and the experimental regimen is HFRTsib. The CFRTseq delivers 50 Gy in 25 fractions to the whole breast with or without regional lymph node in 5 weeks and sequential tumor bed boost of 10 Gy in 5 fractions in 1 week. The HFRTsib delivers 40 Gy in 15 fractions to the whole breast with or without regional lymph node in 3 weeks and SIB of 48 Gy in 15 fractions to the tumor bed. The schematic overview of the study design is shown in Fig. 1. The study protocol was approved by the Institutional Review Board of Fudan University Shanghai Cancer Center and all methods were performed in accordance with the relevant guidelines and regulations.

### Endpoints

#### Primary endpoint

The primary endpoint of this study is locoregional recurrence (LRR), which is defined as recurrences in ipsilateral breast, axillary, supraclavicular nodes and internal mammary nodes.

#### Secondary endpoints

Disease-free survival (DFS), overall survival (OS), incidences of acute and late adverse events, cosmetic outcomes and quality of life (QoL) will be compared between the two groups of HFRTsib and CFRTseq.

### Eligibility criteria

#### Inclusion criteria

1. Female patients aged between 18 and 70 years.
2. Single tumor or multiple tumor that could be removed by one quadrantectomy.
3. Receiving BCS with negative surgical margins.
4. Receiving sentinel lymph node biopsy and/or axillary lymph node dissection. If sentinel lymph node is positive, axillary lymph nodes should be dissected at stations I and II. The total number of nodes dissected should be at least ten.
5. Tumor bed can be outlined by metal markers placed during surgery or postoperative changes can be clearly identified on CT.
6. Histology confirmed invasive breast cancer with pathological classified T1 ∼ 3N0 ∼ 3M0.
7. The status of estrogen receptor, progesterone receptor, human epidermal growth factor receptor 2 and Ki-67 were determined by immunohistochemistry.
8. No distant metastasis before radiotherapy.
9. No history of serious medical disease or other serious comorbidities, such as psychiatric and allergy history.
10. Karnofsky performance score (KPS) of 80 or higher.
11. Provision of written informed consent.

#### Exclusion criteria

1. T4 disease or distant metastasis.
2. Suspected or confirmed supraclavicular/internal mammary lymph node metastasis detected pre-operation or pre-radiation.
3. Histology confirmed ductal carcinoma in situ without invasive cancer.
4. Bilateral breast cancer.
5. Receiving neoadjuvant systemic treatment.
6. Multiple tumor that could not be removed by one quadrantectomy.
7. Suspected malignant lesion in the ipsilateral or contralateral breast detected by imaging examination without pathological confirmation of benign.
8. KPS of 70 or less.
9. Active infection present.
10. History of severe diseases or mental illness.
11. Prior history of malignancies, except for non-malignant melanoma skin cancer and cervix carcinoma in situ.
12. Prior history of ipsilateral breast or regional nodal area irradiation.
13. Pregnant or lactating.
14. Not expected to complete the treatment because of poor compliance or critical disease.

### Randomization

Participants with informed consent will be checked for eligibility before randomization. Eligible patients will be randomized into the HFRTsib group and the CFRTseq group in a ratio of 1:1. Stratified block randomization method will be applied and randomization will be performed with a randomization software designed by statisticians. The stratification factors of the randomization include age (≥ 50 vs. <50), ER status (positive vs. negative), axillary lymph node status (positive vs. negative) and adjuvant chemotherapy (yes vs. no).

### Radiotherapy

#### Target volumes definition

Post-lumpectomy clinical target volumes (CTVs) include whole breast and post-operative tumor bed. When indicated, regional lymph nodes are also included. Target volume definitions are indicated below (Table 1) [20]. Gross target volume (GTV) of tumor bed should be contoured according to metal markers (usually titanium clips) placed during surgery and/or postoperative changes such as seroma. For patients without metal markers, postoperative changes should be clearly identified through simulation computed tomography (CT) and ultrasound. CTV of tumor bed is expanded from tumor bed GTV by 1 cm and is limited to the whole breast CTV. The planning target volumes (PTV) of whole breast and tumor bed should be expanded from CTV by 0.5 cm in all directions and be limited to 0.5 cm beneath the skin surface. The regional nodal area will be irradiated in patients with pathologically positive axillary lymph nodes either detected in sentinel lymph node biopsy or in axillary lymph node dissection. CTVs of the regional lymph nodal area include the undissected axilla (principally axillary level II and III), the supraclavicular nodes, and the internal mammary nodes. PTVs are expanded by 0.5 cm around the regional lymph node CTV.

**Table 1:** Guidelines of CTV delineation

CTV	Cranial	Caudal	Medial	Lateral	Ventral	Dorsal
Breast	Upper border of palpable/visible breast tissue; up to the inferior edge of the sternoclavicular joint	Most caudal CT slice with visible breast/mammary fold	Medial border of palpable/visible breast tissue; maximally to the parasternal line	Lateral border of visible/palpable mammary gland	0.5 cm beneath skin surface (including visible breast tissue)	Anterior edge of the pectoralis major/intercostal muscles/ribs
Axillary level I	0.5 cm cranial to axillary vein	Level of 4th anterior rib, including postoperative change of SLNB	Axillary level II; chestwall	The line between pectoralis major and deltoid/ latissimus dorsi	Pectoralis major; pectoralis minor	Thoracodorsal vessels; the line between intercostal muscles and deltoid/ latissimus dorsi
Axillary level II	Cranial edge of axillary artery	Caudal edge of pectoralis minor	Medial border of pectoralis minor	Lateral border of pectoralis minor	Posterior surface of pectoralis minor	0.5 cm dorsal to axillary vein; anterior edge of the intercostal muscles
Axillary level III	0.5 cm cranial to subclavian vein	0.5 cm caudal to subclavian vein	Junction of subclavian and internal jugular veins	Medial border of the pectoralis minor	Pectoralis major	0.5 cm dorsal to axillary vein; anterior edge of the intercostal muscles
INT	0.5 cm cranial to subclavian vein	Caudal border of CTV axillary level II	Medial border of pectoralis minor	Lateral border of pectoralis minor	Pectoralis major	Pectoralis mimor
SCN	Caudal edge of cricoid cartilage	Junction of subclavian and internal jugular veins	Medial edge of the internal jugular vein, excluding thyroid and the common carotid artery	Sternocleidomastoid; the junction of clavicle and 1st rib	Medial edge of the sternocleidomastoid/clavicle	Ventral edge of the scalene muscle bundles
IMN	0.5 cm caudal to subclavian vein	Cranial edge of 4th rib	0.5 cm medial to the internal thoracic vessels	0.5 cm lateral to the internal thoracic vessels	Ventral edge of the internal thoracic vessels	Parietal pleura

CTV: clinical target volume; INT: interpectoral lymph nodes; SCN: supraclavicular lymph nodes; IMN: internal mammary node; SLNB: sentinel lymph node biopsy

#### Normal tissue contouring

Contralateral breast, bilateral lung, heart, thyroid, ipsilateral humeral head, cervical esophagus and brachial plexus are contoured in each patient.

#### Dose prescription

For CFRTseq group, the prescribed dose of PTV is 50 Gy in 25 fractions over five weeks and sequential tumor bed boost of 10 Gy in 5 fractions, 2 Gy per fraction. For HFRTsib group, the prescribed dose of PTV is 40 Gy in 15 fractions over three weeks, 2.67 Gy per fraction. Tumor bed of PTV will receive simultaneous boost, with total dose of 48 Gy in 15 fractions, 3.2 Gy per fraction.

#### Treatment planning and dose constraints

CT-based treatment plan and tissue inhomogeneity correction is required. Inversely IMRT or volumetric modulated arc therapy treatment plans are both allowed. It is required that at least 95% of PTV volume should receive 95% of the prescribed dose. Dose to heart in every treatment plan should be as low as possible. The detailed dose constraints of treatment planning are listed in Table 2. Position verification will be performed before treatment and at least once a week during treatment with electronic portal imaging device or cone beam computed tomography. Setup errors less than 5 mm is allowed.

**Table 2:** Dose-volume constraints of treatment planning

Structure	CFRTseq				HFRTsib			
	WBI		WBI + RNI		WBI		WBI + RNI	
	Per protocol	Alteration allowed	Per protocol	Alteration allowed	Per protocol	Alteration allowed	Per protocol	Alteration allowed
Breast PTV	V47.5 ≥ 95%	V45 ≥ 90%	V47.5 ≥ 95%	V45 ≥ 90%	V38 ≥ 95%	V36 ≥ 90%	V38 ≥ 95%	V36 ≥ 90%
	Dmax ≤ 57.5 Gy	Dmax ≤ 60 Gy	Dmax ≤ 57.5 Gy	Dmax ≤ 60 Gy	Dmax ≤ 46 Gy	Dmax ≤ 48 Gy	Dmax ≤ 46 Gy	Dmax ≤ 48 Gy
Boost PTV	V57 ≥ 95%	V54 ≥ 90%	V57 ≥ 95%	V54 ≥ 90%	V45.6 ≥ 95%	V43.2 ≥ 90%	V45.6 ≥ 95%	V43.2 ≥ 90%
	Dmax ≤ 69 Gy	Dmax ≤ 72 Gy	Dmax ≤ 69 Gy	Dmax ≤ 72 Gy	Dmax ≤ 55.2 Gy	Dmax ≤ 57.6 Gy	Dmax ≤ 55.2 Gy	Dmax ≤ 57.6 Gy
Axillary PTV	-	-	V47.5 ≥ 95%	V45 ≥ 90%	-	-	V38 ≥ 95%	V36 ≥ 90%
	-	-	Dmax ≤ 57.5 Gy		-	-	Dmax ≤ 46 Gy	
SCN PTV			V47.5 ≥ 95%	V45 ≥ 90%			V38 ≥ 95%	V36 ≥ 90%
IMN PTV	-	-	V45 ≥ 95%	V40 ≥ 90%	-	-	V36 ≥ 95%	V32 ≥ 90%
	-	-	Dmax ≤ 57.5 Gy		-	-	Dmax ≤ 46 Gy	
Ipsilateral lung	V20 ≤ 15%	V20 ≤ 20%	V20 ≤ 30%	V20 ≤ 35%	V17 ≤ 15%	V17 ≤ 20%	V17 ≤ 30%	V17 ≤ 35%
Contralateral lung	V5 ≤ 10%		V5 ≤ 15%		V4.2 ≤ 10%		V4.2 ≤ 15%	
Heart (left-sided)	Dmean ≤ 4 Gy	Dmean ≤ 5 Gy	Dmean ≤ 6 Gy	Dmean ≤ 8 Gy	Dmean ≤ 3.4 Gy	Dmean ≤ 4.2 Gy	Dmean ≤ 5.1 Gy	Dmean ≤ 6.8 Gy
Heart (right-sided)	Dmean ≤ 2 Gy	Dmean ≤ 2.5 Gy	Dmean ≤ 2.5 Gy	Dmean ≤ 3 Gy	Dmean ≤ 1.6 Gy	Dmean ≤ 2 Gy	Dmean ≤ 2 Gy	Dmean ≤ 2.5 Gy
Contralateral breast	V5 ≤ 10%		V5 ≤ 10%		V4.2 ≤ 10%		V4.2 ≤ 10%	
Thyroid	-	-	V30 ≤ 50%	Dmean ≤ 25 Gy	-	-	V25 ≤ 50%	Dmean ≤ 21 Gy
Humeral head	-	-	V30 ≤ 30%	Dmean ≤ 25 Gy	-	-	V25 ≤ 30%	Dmean ≤ 21 Gy

HFRTsib: hypofractionated radiotherapy with simultaneously integrated boost; CFRTseq: conventional fractionated radiotherapy with sequential boost; WBI: whole breast irradiation; RNI: regional node irradiation; SCN: supraclavicular lymph nodes; IMN: internal mammary node; Dmax: maximum dose; Dmean: mean dose: Vx: percentage of volume receiving dose of x Gy

### Follow-up

Regular follow-up visits will be scheduled every three months during the first year after RT, every six months for two to five years after RT, and annually thereafter. Acute radiation toxicity will be evaluated before, during, at the end of, and three months after RT. Late radiation toxicity will be assessed before RT, three months after RT, and then annually. Acute toxicity will be graded according to the Cancer Institute’s Common Terminology Criteria for Adverse Events version 4.03 (CTCAE 4.03) and the Radiation Therapy Oncology Group (RTOG) radiation morbidity scale. Late toxicity will be evaluated primarily based on the RTOG late radiation morbidity scale and the LENT-SOMA scale [21, 22]. Cosmetic outcomes will be scored by patients, research nurses and physicians respectively using the Harvard Scale system [23]. QoL assessment will be performed using EORTC-QLQ-C30 and QLQ-BR23 questionnaires. All the case report forms will be documented by the researchers and those who were authorized by the researchers. The case report forms and other related data will be preserved for at least five years after the completion of the trial.

### Statistics

#### Sample size calculation

The current trial was designed to verify the non-inferiority of HFRT combined with SIB compared to CFRT combined with sequential boost in patients undergoing BCS. The primary endpoint of the trial is LRR. Based on START A and B, the five-year LRR was 2.2%∼5.2% among patients treated with HFRT [8, 9]. In reference to WBI among Chinese population, a recent study comparing whole-breast HFRT and CFRT reported that the five-year LRR was 3.8% and 3.1% in CFRT and HFRT groups, respectively [24]. In our center, the five-year LRR in WBI patients without neoadjuvant systemic therapy was 2.8% [25]. Therefore, the five-year LRR of the CFRT group in the current trial is assumed to be 3% and the five-year LRR of the HFRT group is expected to be less than 5% (Hazard ratio [HR] of 1.68). With a study power of 80%, one-sided alpha value of 0.025, four-year recruitment period and five-year follow-up, at least 1383 participants will be required for each group, and 2766 participants required in total. Taking the withdrawal rate as 5%, the sample size will be 2904 (1452 for each group). The sample size was calculated using PASS2008.

#### Statistical analysis

The primary endpoint LRR will be estimated with the cumulative incidence method. Distant metastasis-free survival, DFS and OS will be calculated using Kaplan-Meier method. Differences of the survival outcomes between HFRTsib and CFRTseq groups will be compared with the log-rank test and analyzed by the COX regression model. Acute and late toxicities will be described as frequency and severity. Comparison of toxicities and cosmetic results between the two groups will be performed with the Chi-square test.

## Discussion

In this report, we present a phase III multi-centered, randomized controlled trial that aims to compare local control, survival, toxicities and cosmetic results after HFRT combined with SIB and CFRT with sequential boost in patients receiving BCS.

The lumpectomy cavity region has been proven to have a high risk of recurrence, especially in younger women and those with high grade tumors [26]. Previous trials from Canada and the START series trials have shown the safety and effectiveness of hypofractionated WBI combined with a sequential boost to the lumpectomy cavity [7, 10, 27]. It is worth noting that the onset of breast cancer in the Chinese population occurs approximately ten years earlier than in western countries. Thus, the importance of tumor bed boost may be even greater for breast cancer patients in China. Traditionally, the boost is delivered sequentially, requiring an additional 4 ∼ 8 fractions [26, 28]. However, dose escalation to the tumor bed by SIB has been proposed for several reasons. First, hypofractionated SIB takes advantage of the low α/β value of breast cancer, leading to a biological advantage. Additionally, SIB provides better conformity and homogeneousness of dose distribution, as well as a shorter treatment duration [29]. The IMRT-MC2 trial was a randomized trial comparing a sequential boost of 16 Gy in 8 fraction to the tumor bed and a SIB of 64.4 Gy to tumor bed combined with WBI of 50.4 Gy in 28 fractions. The results showed no differences in local control or long-term adverse effects between sequential and simultaneous boost [16]. With regards to the application of SIB in hypofractionated WBI, the IMPORT HIGH trial evaluated two different hypofractionated concurrent boost regimens. The control arm of the trial received 40 Gy in 15 fractions to the whole breast and a sequential tumor-bed boost of 16 Gy in 8 fractions. The two experimental arms received 36 Gy in 15 fractions to the whole breast, with different fractionation regimens for the concurrent tumor-bed boost. One experimental arm received a dose of 48 Gy in 15 fractions, while the other received 53 Gy in 15 fractions. After a follow-up of 74 months, the experimental arm with a dose of 48 Gy/15 fractions demonstrated non-inferior local control and adverse effects compared to the control arm. The 5-year ipsilateral breast tumor relapse (IBTR) rates for the control and experimental arm were 1.9% and 2.0% respectively, and the incidences of moderate or marked breast induration for the two arms were 11.5% and 10.6% respectively (*p* = 0.40). On the other hand, the experimental arm with a dose of 53 Gy/15 fractions showed worse local control and an increased incidence of breast induration. Overall, this trial demonstrated the safety and efficacy of a dose of 48 Gy for the simultaneous tumor-bed boost [17].

Another randomized trial concerning SIB in the context of HFRT is RTOG 1005, which compares SIB of 48 Gy/15 fractions with conventional sequential boost in breast-conserving patients [30]. The result of the study is pending. The trial HYPOSIB also discuessed SIB of 48 Gy/15fx in patients after BCS. The preliminary results were presented during the 2020 American Society of Clinical Oncology annual meeting, and initially proved the safety of HFRT with SIB technique [31]. In our center, a phase II trial investigating SIB technique irradiated the whole breast with 45 Gy in 25 fractions, and the dose to the tumor bed was simultaneously escalated to 60 Gy. The 5-year locoregional control rate for patients with invasive breast cancer was 98.7%, and none of the patients had grade 3 skin toxicity [32]. Our phase II trial with respect to HF-WBI combined with SIB in early breast cancer patients employed a total dose of 48 Gy to the tumor bed and 40 Gy to the whole breast, delivered in 15 fractions over the span of three weeks. The results showed well tolerance and favorable cosmetic outcomes, with no grade 3 radiodermatitis observed and an 81.8% satisfaction rate among patients regarding breast cosmesis. Two-year DFS rate was 98.6% [19]. Based on previous results, we will employ SIB in the HFRT arm compared with CFRTseq, the fractionation scheme used was the same as our phase II trial.

In the current trial, patients with positive axillary lymph nodes will receive RNI, and all the CTVs of RNI include IMN. According to EORTC 22,922 and MA. 20, RNI could significantly improve locoregional control in breast cancer patients with positive axillary nodes [33, 34]. Recent studies suggested that patients with medial/central tumors are likely to benefit from IMN irradiation (IMNI) [35, 36]. However, due to potential cardiac toxicity, the administration of IMNI remains controversial. The IMN region was not irradiated in previous trials regarding HFRT after BCS. In the current trial, IMNI will be delivered in all the patients receiving RNI. Dose to the heart should be strictly constrained and techniques such as deep inspiration breath hold (DIBH) will be applied to minimize heart dose. Major cardiovascular events will be documented during follow-up and early cardiac damage will be monitored through electrocardiography and echocardiography. Long-term results of our trial are expected to provide important data for the safety of hypofractionated IMNI.

The ongoing trial is a multi-centered, prospective randomized phase 3 study which intends to recruit 2904 participants. The study protocol version 1.0 has been approved by the ethics committee of Fudan University Shanghai Cancer Center in April 2018 and the latest modification of the protocol was made in January 2021. This trial is registered in ClinicalTrails.gov (NCT04025164). Recruitment of the trial started from June 2018 and is anticipated to be completed by June 2024. 90% of the participants are expected to be recruited from our center and 10% are expected to be from other participating centers. The follow-up time of the trial will be at least five years. The results of the present trial are expected to provide important evidence for the effectiveness and tolerance of short-course radiotherapy in breast cancer patients after BCS in the age of modern treatment strategies.

## Conclusion

The aim of the presented trial is to testify the non-inferiority of HFRT combined with SIB compared to CFRT with a sequential boost in women with breast cancer after BCS. Locoregional control, long-term survival, toxicity, and cosmetic outcomes will be evaluated. The non-inferiority of HFRT combined with SIB will support the application of a shorter-course, lower-cost radiotherapy scheme for breast cancer patients. Therefore, the anticipated results of this trial will provide high-level evidence to inform the clinical decision-making process for adjuvant radiotherapy after BCS.

### Electronic supplementary material

Below is the link to the electronic supplementary material.

Supplementary Material 1

## Data Availability

No datasets were generated or analysed during the current study.

## Abbreviations

ASTRO: American Society for Radiation Oncology
BCS: Breast-conserving surgery
CFRTseq: Conventional fractionated radiotherapy with sequential boost
CT: Computed tomography
CTV: Clinical target volume
DFS: Disease-free survival
DIBH: Deep inspiration breath hold
GTV: Gross target volume
HFRTsib: Hypofractionated radiotherapy combined with simultaneous integrated boost
IBTR: Ipsilateral breast tumor relapse
IMNI: Internal mammary node irradiation
IMRT: Intensity modulated radiation therapy
KPS: Karnofsky performance score
LRR: Locoregional recurrence
PTV: Planning target volume
QoL: Quality of life
RNI: Regional node irradiation
WBI: Whole breast irradiation
